# Other duties as required: efficient use of human resources in Mozambique

**DOI:** 10.1186/2052-3211-7-S1-O26

**Published:** 2014-12-17

**Authors:** Wendy Prosser, Ruth Bechtel

**Affiliations:** 1 VillageReach, Seattle, WA, USA; 2 VillageReach, Maputo, Mozambique

## Background

The crisis in human resources for health in low-income countries has been documented many times over by research and experience. A fundamental issue in human resources in the vaccine supply chain is the system in which the health worker is working, which requires more than training and revised guidelines to address.

## Method

To improve vaccine supply chain management in Mozambique a Dedicated Logistics System (DLS) was trialled. This system works through providing dedicated personnel to consolidate supply chain functions at the provincial level where limited resources are more likely to be available. These dedicated personnel distribute vaccines directly to health centres based on actual consumption, collects data, provides supportive supervision and cold chain preventive maintenance.

## Results

With the DLS, supply chain tasks are consolidated to a few dedicated personnel, using two to three vehicles and the corresponding resources to achieve direct delivery to all health centers in order to achieve higher vaccine coverage rates. For comparison, a multi-tiered system which follows standard administrative levels requires a vehicle, driver and vaccine specialist at each level, and about 100 health centre staff who perform supply chain tasks as a minimum part of their overall responsibilities. Total equipment and human resource requirements is 11 vehicles, the accompanying fuel, and more than 130 personnel who are adequately trained and skilled in supply chain management.

## Discussion

Engaging dedicated, trained logisticians to manage supply chain functions requires less forecasting skills from health workers and frees up their time to focus on patient care. As such, training and provision of technology can be focused on these specialists. The placement of these personnel matches the reality of the system as financial resources required for distribution are more likely to be available at the provincial level than the district level. An estimated 138 staff days/month per province are required for logistics duties with the DLS, compared to 348 staff days/month for a multi-tiered system.

## Lessons learned

The effectiveness of dedicated personnel is largely due to its synergy with the overall system which has been specifically adapted to the on-the-ground realities of these provinces in Mozambique. Dedicated positions were created to fit the context, available resources at the appropriate levels, and the system design itself.

